# Pulmonary vascular Ehlers-Danlos syndrome with hemoptysis as the main manifestation: CT and histologic findings of lung parenchymal damage

**DOI:** 10.1186/s13023-025-04113-4

**Published:** 2025-11-21

**Authors:** Yongxia Lei, Gengfeng Chen, Tao Chen, Gengjia Chen, Haiyan Xu, Xilai Chen, Jin Zhao, Cheng Hong, Yu Deng, Xinchun Li

**Affiliations:** 1 Department of Radiology, The First Affiliated Hospital, Guangzhou Medical University, Guangzhou, China; 2 Guangzhou Medical University, Guangzhou, China; 3 Department of Pathology, The First Affiliated Hospital, Guangzhou Medical University, Guangzhou, China; 4 State Key Laboratory of Respiratory Diseases, Guangdong Key Laboratory of Vascular Diseases, Guangzhou Institute of Respiratory Health, The First Affiliated Hospital of Guangzhou Medical University, Guangzhou, China

**Keywords:** Ehlers-Danlos syndrome, Hemoptysis, Hemothorax, Pneumothorax, CT

## Abstract

**Background:**

Vascular Ehlers-Danlos syndrome (vEDS) is a rare inherited connective tissue disease caused by mutations in the COL3A1 gene. The disease can cause fatal complications such as rupture of the arteries, uterus, and intestine, as well as pulmonary complications, including spontaneous pneumothorax and hemoptysis. Since hemoptysis in vEDS is rare and often misdiagnosed, this study aims to summarize its clinical features, CT findings and the diagnosis and treatment process, thereby improving clinical understanding.

**Methods:**

Patients with vEDS presenting with hemoptysis treated at the First Affiliated Hospital of Guangzhou Medical University from May 2017 to December 2024 were collected. Inclusion criteria included hemoptysis, chest CT, and COL3A1 gene test results. The clinical manifestations, CT and pathological data of the patients were collected.

**Results:**

The cohort included eight males and one female (mean age 22.22 ± 5.72 years). All patients presented with recurrent hemoptysis and cough. The CT findings included patchy ground-glass opacities, fibrous cords, nodules, and cavities, which were predominantly located in the lower lungs. 5 cases showed diffuse erythrocyte exudation and increased hemosiderin-laden macrophages. Eight cases were initially misdiagnosed as infectious diseases, of which 4 cases were treated with diagnostic anti-tuberculosis therapy.

**Conclusion:**

This study demonstrates that the clinical and imaging manifestations in vEDS patients presenting with hemoptysis lack specificity and are frequently misdiagnosed as infectious diseases. For young men with repeated hemoptysis and persistent CT abnormalities, especially when anti-infective treatment proves ineffective, vEDS should be considered and early genetic testing should be performed. Early diagnosis is crucial to prevent severe complications.

**Clinical trial number:**

Not applicable.

**Supplementary Information:**

The online version contains supplementary material available at 10.1186/s13023-025-04113-4.

## Introduction

Ehler-Danlos syndrome (EDS) is a rare group of inherited connective tissue disorders, comprising 14 types that primarily affect the joints and skin, with symptoms including joint relaxation, joint pain, skin softness, and abnormal scarring [1–3]. Among them, vascular Ehlers-Danlos syndrome (vEDS) is caused by pathogenic variations of COL3A1 gene, which can lead to increased arterial and organ fragility and early death [4, 5]. Fatal complications such as ruptured arteries, uteri, and intestines may occur, and aneurysms or dissections may precede arterial rupture [6, 7]. Studies have shown that type III collagen is expressed in both pulmonary vascular and parenchymal fibroblasts [8]. When COL3A1 gene expression is defective, the integrity of pulmonary vascular and parenchymal structures will be affected, resulting in complications such as spontaneous pneumothorax, hemopneumothorax and pulmonary hemorrhage. Frank et al. collected 144 genetically confirmed vEDS patients from the French National Referral Center for Rare Vascular Diseases, among which 74 (51.4%) had arterial events, 33 (22.9%) had gastrointestinal events, and 15 (10.4%) had pulmonary events [5]. Bowen et al. collected 180 vEDS patients from the center of UK National Diagnostic Service and reported similar results, including 72 (40%) vascular events, 25 (13.88%) intestinal perforations, and 10 (5.55%) pneumothoraxs [9].

When vEDS is involved in the lungs, CT can show emphysema (mostly centrilobular or septal type), pulmonary bullae, pulmonary air sacs, cavities, fibrous nodules, and ground-glass density. A recent study of 136 patients with vEDS revealed that 78 (57.4%) had lung abnormalities, the most common being emphysema (44 cases, 32.3%), most of which may be asymptomatic [10]. Other abnormalities include striated shadows, tufted micronodules, and cavitary nodules. The most common complications of vEDS lung involvement are spontaneous pneumothorax and hemopneumothorax, while hemoptysis is rare. In Frank’s study, only 3 patients (2.2%) showed hemoptysis [5], and only 5 of 180 patients with lung disease in Sheffield Center in the United Kingdom showed hemoptysis symptoms [9]. It can be seen that vEDS with pulmonary complication is a rare presentation, and most of the literature consists of case reports [11–20]. The causes of hemoptysis are diverse, including infection, tumor, bronchiectasis and pulmonary vascular disease, and lack of understanding of pulmonary vEDS often leads to incorrect diagnosis [21]. When the patient is in the active bleeding stage, CT can show ground glass density focus with cavitary nodules and nodules with halo sign, which is easily misdiagnosed as infectious lesions. The stable stage is characterized by fibrous cords and solid nodules, which can be easily judged as changes after inflammation. These signs are often coexisting, increasing the difficulty of diagnosis for clinicians and radiologists. Pulmonary symptoms of vEDS often precede other fatal complications such as arterial rupture, uterine rupture in pregnancy, and rupture of the small intestine [22].

Therefore, this study aims to summarize the clinical manifestations, CT features, pathological features, diagnosis and treatment process of vEDS with hemoptysis as the main symptom, so as to improve the understanding of clinicians and radiologists on the disease, promote early diagnosis and standardized management, and prevent the occurrence of more serious complications.

## Methods

### Patient characteristics

In this study, patients with suspected pulmonary vEDS who were admitted to the First Affiliated Hospital of Guangzhou Medical University from May 2017 to December 2024 were retrospectively collected by electronic medical record system. Inclusion criteria included: (1) Presence of hemoptysis with or without pneumothorax or hemopneumothorax; (2) Had chest CT examination data (including optional enhanced CT, pulmonary artery CTA, bronchial artery CTA; (3) Positive COL3A1 genes test result. Genetic testing was performed with variant interpretation conducted following the American College of Medical Genetics and Genomics (ACMG) guidelines. Only pathogenic or likely pathogenic variants in COL3A1 were considered diagnostic. The clinical data collected included demographic characteristics, clinical symptoms (frequency, quantity, character, chest pain, pneumothorax, etc.), skin and joint manifestations, family history, date of onset and diagnosis, and follow-up data. This study was approved by the Ethics Committee of the First Affiliated Hospital of Guangzhou Medical University.

### CT protocol and image acquisition

All patients underwent one or more spiral CT examinations (SOMATOM Definition AS + 128-slice, SIEMENS), including plain scan, enhanced scan, pulmonary CTA, and bronchial artery CTA. Scanning parameters: tube voltage 120 kVp, tube current automatic modulation (90–250 mAs), collimation 0.75 –2.0 mm, pitch 0.8-1.0, layer thickness/pitch 2 mm/2 mm, matrix 512 × 512, FOV 400 mm. The enhanced scan was performed using an intravenous infusion of 60% iodine contrast agent (1.2-1.5 ml/kg, 3.0-3.5 ml/s) with a high-pressure syringe. Threshold triggering method was used for CTA, and the ROI thresholds for the main pulmonary artery and descending aorta were 100HU and 200HU, respectively. The images were reconstructed by bone algorithm and standard algorithm and then transmitted to PACS system for coronal, sagittal and MIP reconstruction.

### CT and PET-CT image analysis

CT images were reviewed by a radiologist with 15 years of experience specializing in pulmonary vascular imaging. Pulmonary imaging features were documented for all patients, including ground-glass opacities, fibrotic strands, consolidation, non-cavitary nodules, cavitary nodules, bullae, emphysema, pneumothorax, pleural effusion, pleural thickening, pulmonary artery embolism, bronchial artery abnormal dilation, and the maximum SUV values of 18FDG-labeled PET-CT examination. The location of lesions and their evolution during follow-up were also recorded.

### Pathological examination

All pathological specimens, obtained through surgical resection, or needle biopsy, were collected for analysis. The specimens were subjected to routine fixation, HE staining and immunohistochemical staining, and were analyzed by a pathologist with 15 years of experience in pulmonary pathology. Descriptive statistical methods were used to analyze the pathological data.

## Results

### Clinical characteristics and baseline demographics

Table 1 summarized the clinical characteristics of 9 cases of vEDS with pulmonary hemoptysis. From May 2017 to December 2024, a total of 13 patients with suspected pulmonary vEDS were treated in our hospital. 3 patients with negative COL3A1 gene test indicating vEDS changes were excluded, and 1 patient with positive COL3A1 gene test but no hemoptysis was excluded. Finally, 9 cases were included in the study, comprising 8 males and 1 female. The mean age was 22.22 ± 5.72 years and the mean BMI was 23.57 ± 3.83 years. The follow-up time of 9 patients was 0.57 to 6.83 years (mean 2.78 ± 2.08 years). Except for one case of acute hematemesis for half a day, the other cases had a long history, ranging from half a year to more than 5 years, and had many visits to other hospitals. All the patients presented with repeated cough and hemoptysis, and 1 of them had hematemesis as the initial symptom, and upper gastrointestinal hemorrhage was ruled out by emergency gastroscopy, whose blood was bright red. 7 cases had blood clots and 2 cases had blood in the sputum. The daily amount of hemoptysis was about 10-50 ml, of which 3 cases had paroxysmic massive hemoptysis, up to 100-200 ml. Among the 9 cases, family members of 2 patients were identified as asymptomatic mutation carriers through genetic testing.

**Table 1:** Clinical characteristics and baseline demographics of patients with pulmonary hemorrhage-Type vEDS

Clinical Characteristic	Pulmonary vEDS
Age, years	22.22 ± 5.72
Male	8(89%)
Body mass index, kg/m2	23.57 ± 3.83
Smoking status	0
Hemoptysis	
Blood clots	7 (78%)
Blood-streaked sputum	2 (22%)
Bright red appearance	9 (100%)
Daily volume (10–50 mL/day)	9 (100%)
Massive hemoptysis (100–200 mL/episode)	3 (33%)
Associated Symptoms	
Chest pain (with recurrent bilateral pneumothorax/effusion)	2 (22%)
Fever	1 (11%)
Skin manifestations	3 (33%)
Joint manifestations	0
Family History	2
Brochoscopy	
No active bleeding	8
Active bleeding	1
Bronchoalveolar lavage fluid	
Negative	6
Positive	3
Time from symptom onset to vEDS diagnosis (years)	2.19 ± 1.77

Two patients presented with chest pain combined with bilateral recurrent fluid pneumothorax. One patient had fever. 3 cases were accompanied by skin changes, 1 case showed systemic skin thinning, 1 case showed systemic skin multiple scars, and 1 case showed hyperelasticity. No joint changes were observed. The time from onset of symptoms to diagnosis of vEDS ranged from 0.36 to 5.07 years, with an average of 2.19 ± 1.77 years. All 9 patients underwent bronchofiberscopy and alveolar lavage fluid examination, with one case showing active bleeding under bronchoscopy, while the remaining patients showed no abnormal changes. In 3 cases, NGS could detect a variety of pathogenic bacteria, including Haemophilus influenzae, novel coronavirus, human herpes virus, influenza A virus, pseudomonas aeruginosa, Streptococcus constellatus, and mycoplasma pneumoniae, while no pathogenic bacteria were detected in 6 cases.

### CT features

**Fig. 1:**
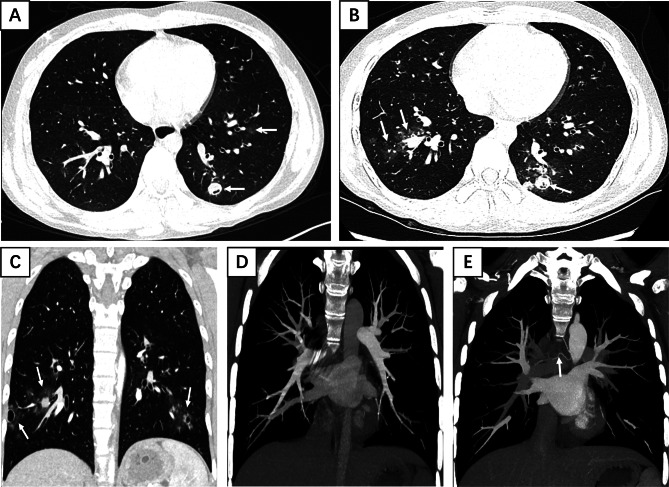
A 16-year-old male with recurrent hemoptysis for over one year, recent exacerbation for 10 + days and fever for 5 days. Previous bronchoalveolar lavage detected Haemophilus influenzae but anti-infection therapy was ineffective. (**A**) Emergency chest CT (lung window) on January 9, 2024, showed scattered patchy ground-glass opacities and cavitary nodules (white arrows) in both lungs, with smooth nodular margins and minimal air lucency within the nodules. (**B**-**C**) CT re-examination two days later when symptoms worsened, revealed increased patchy ground-glass opacities, cavitary and non-cavitary nodules (white arrows) in both lungs, with ground-glass halos surrounding the nodules. (**D**) Pulmonary CTA with MIP reconstruction showed no evidence of embolism. (**E**) Bronchial artery CTA with MIP reconstruction demonstrated right bronchial artery diameter of approximately 2 mm and left of 1.3 mm, without abnormal dilation. Dermatological examination revealed thin skin; COL3A1 genetic testing was positive, confirming vEDS diagnosis

Table 2 summarizes the demographic characteristics, diagnostic timeline, CT examination protocols, and clinical management of vEDS patients with pulmonary hemoptysis. Before diagnosis of vEDS, the number of chest CT plain scans in 9 patients ranged from 3 to 12 times, with a mean of 6.22 ± 3.15 times (including CT examinations performed at both our hospital and other hospitals, confirmed through telephone follow-up). The number of contrast-enhanced CT scans ranged from 1 to 3 times, with a mean of 1.67 ± 1 times. Because the patients had hemoptysis, 8 of the 9 patients underwent bronchial artery CTA and 6 conducted pulmonary artery CTA (Table 2). The lesions were scattered in the bilateral lungs, and were more common in the lower lungs. CT showed patchy ground-glass opacities in 9 cases, fibrous strip shadow in 8 cases, non-cavernous nodule in 9 cases, cavernous nodule in 7 cases, consolidation in 3 cases, emphysema with pulmonary bullae in 3 cases, calcified nodule in 2 cases, and liquid pneumothorax in 2 cases (Supplemental Table). No pulmonary embolism was found in 6 cases of pulmonary artery CTA (Fig. 1D), and no abnormal thickening of bronchial arteries was found in 8 cases of bronchial artery CTA (Fig. 1E).

**Table 2:** CT Examination, clinical diagnosis and management of pulmonary hemoptysis-type vEDS patients

Case	Sex	Age	Onset Date	Diagnosis Date	Duration to Diagnosis (years)	PCTA	BCTA	CT(*P*/E/PET)	CD	TB-Tx	Mutation
1	Male	25	2017/06/21	2022/05/30	4.9	NP	N	12/3/1	Inf	No	COL3A1
2	Male	20	2018/01/08	2019/09/18	1.7	N	N	9/2/0	Inf	No	COL3A1
3	Female	28	2019/05/01	2024/05/23	5.1	N	N	10/2/0	Inf	No	COL3A1
4	Male	14	2019/09/21	2022/10/01	3.0	NP	N	8/3/1	Inf	Yes	COL3A1
5	Male	19	2022/07/01	2022/11/09	0.4	N	NP	4/0/0	ILD	No	COL3A1
6	Male	16	2023/01/05	2024/01/11	1.0	N	N	5/1/0	Inf	No	COL3A1
7	Male	28	2023/02/01	2024/06/15	1.4	N	N	3/1/1	Inf	Yes	COL3A1
8	Male	30	2023/02/01	2024/09/10	1.6	N	N	6/2/0	Inf	Yes	COL3A1
9	Male	20	2024/05/01	2024/11/24	0.6	NP	N	3/1/0	Inf	Yes	COL3A1

Abbreviations: PCTA = Pulmonary CTA; BCTA = Bronchial Artery CTA; CT(P/E/PET) = the numbers of CT Scans (Plain CT/Enhanced CT/PET-CT); CD = Clinical Diagnosis; TB-Tx = Anti-TB Treatment; NP = Not Performed; N = Normal; Inf = Infection; ILD = Interstitial Lung Disease

**Fig. 2:**
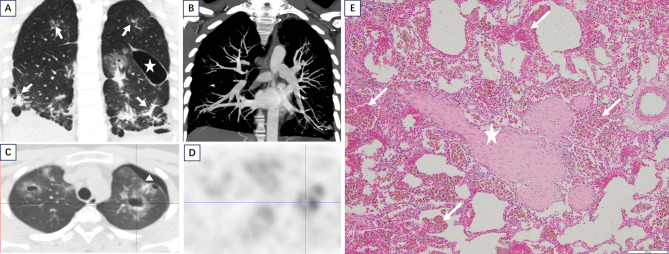
A 14-year-old male with recurrent cough, hemoptysis, and dyspnea for 10 months, with multiple episodes of exercise- or infection-induced pneumothorax. Previous middle lobe biopsy suggested pulmonary hemorrhage. (**A**) Coronal chest CT (lung window) demonstrated bilateral patchy ground-glass opacities, consolidations, irregular solid nodules, linear opacities (white arrows), and pulmonary bulla formation (white asterisk). (**B**) Pulmonary and bronchial artery CTA with MIP reconstruction showed no vascular abnormalities. (**C**) Left-sided pneumothorax was identified (white triangle). (**D**) 18 F-FDG PET-CT (subtraction image) revealed mildly increased uptake (maximum SUV 3.9) at the margins of the left upper lung cavitary lesion and anterior irregular solid nodules. As a metabolic rather than anatomical image, its spatial resolution is thus inherently limited. (**E**) H&E staining (×50) showed fibrous nodules in alveolar septa (white asterisk), with hyaline changes; adjacent alveolar spaces contained diffuse red blood cells and hemosiderin-laden macrophages (white arrows)

**Fig. 3:**
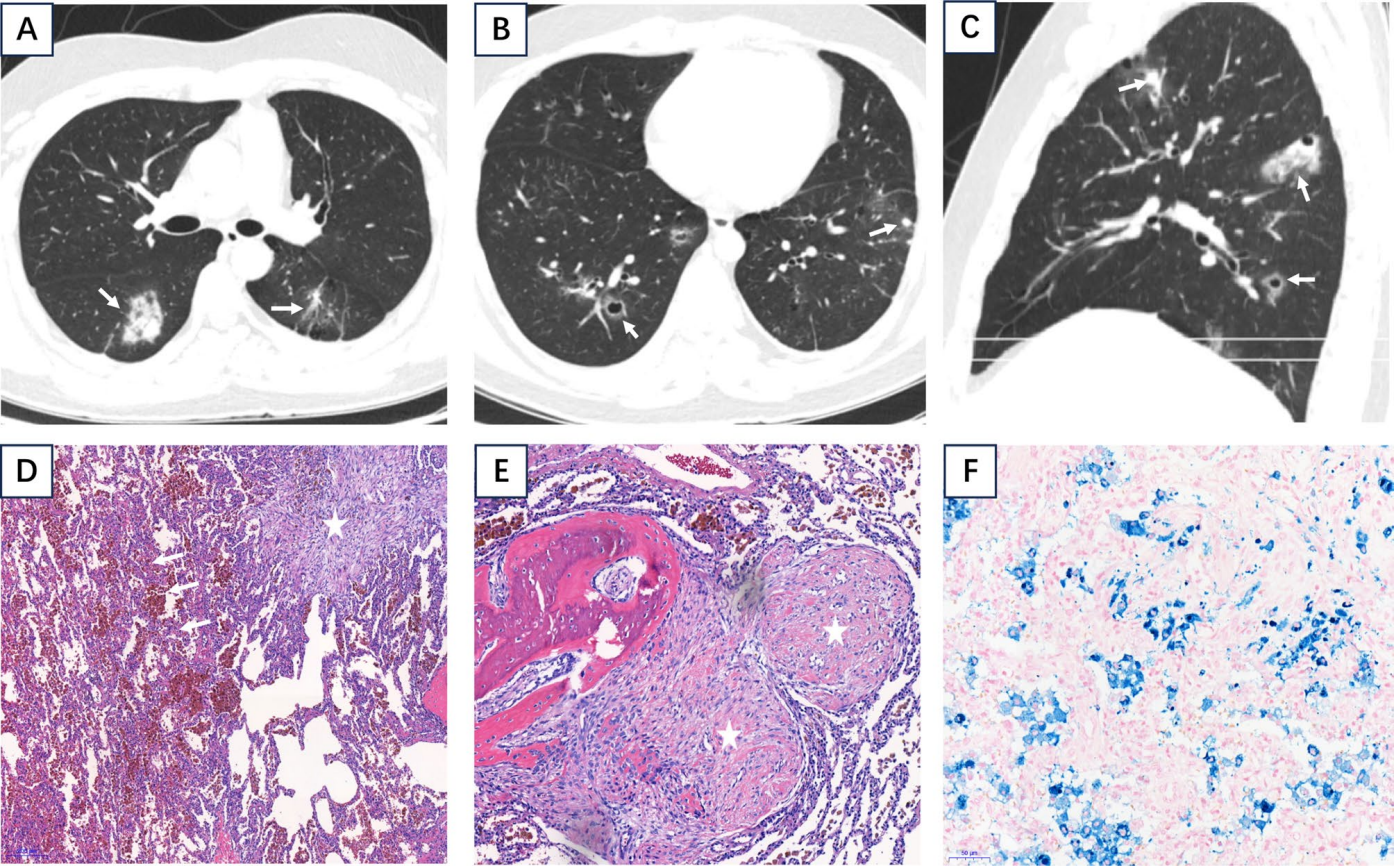
A 20-year-old male with recurrent cough and hemoptysis for six months, with recent exacerbation for 2 days. Previous diagnostic anti-tuberculosis treatment was ineffective. (**A**) Axial chest CT (lung window) demonstrated multiple small patchy consolidations, nodules, and ground-glass opacities in both lungs (white arrows). (**B**) Additional axial CT images showed multiple cavitary and non-cavitary nodules. (**C**) Sagittal CT reconstruction revealed linear opacities and mixed nodular lesions (white arrows). (**D**) H&E staining (×50) showed diffuse red blood cells and hemosiderin-laden macrophages in alveolar spaces. (**E**) Higher magnification H&E staining (×100)demonstrated fibrous nodules in alveolar septa with hyaline changes. (**F**) Iron staining (×200) was positive for hemosiderin

The first CT diagnosis of 8 patients was considered to be infectious lesions. All cases were treated with anti-infective therapy, 4 of which were treated with diagnostic anti-tuberculosis therapy. Symptoms of hemoptysis occurred repeatedly (Table 2). Interstitial lung disease was considered in 1 case. The follow-up time of 9 patients was 0.57 to 6.83 years (mean 2.78 ± 2.08 years) and no other systemic complications occurred. CT re-examination during follow-up showed that the patchy ground-glass density opacity could be absorbed or increased in a short term. Cavitary nodules and non-cavitary nodules could transform into each other. and could enlarge or shrink. Whilst, the fibrous strip shadow, calcified nodules, emphysema, and pulmonary bullae usually had little change. Before diagnosis, three patients underwent PET-CT examinations with 18FDG labeling, and systemic tumor involvement was excluded. Light to moderate uptake of lung lesions was observed (Fig. 2D). The maximum SUV values of lesions were 3.9, 6.5, and 9.2, respectively.

### Pathological features

Among the 9 patients, 4 underwent pulmonary wedge resection, 1 underwent CT-guided puncture biopsy of the left upper pulmonary lesion, and 4 did not undergo pulmonary histopathological biopsy. The specimens from the pulmonary lobar wedge resections of 4 cases measured about 4.2 cm×1.7 cm×1.2 cm, 9 cm×5.5 cm×3 cm, 9 cm×5 cm×2 cm, and 5 cm×6 cm×1.5 cm respectively. Microscopic findings showed diffuse exudation of erythrocytes in alveolar cavity, a large number of hemosiderin cells, and multiple nodules of varying sizes and irregular shapes (Fig. 2A and E). Some were fibroblasts, some were hyaloid nodules, and some were hemorrhagic nodules enclosed by fibrous tissue (Fig. 2C and D). Part of the alveolar septum was ruptured and bulla formed. Immunohistochemical results: CK (-), EMA (+), TTF1 (-), CgA (-), SYN (-), CD56 (+), Vim (+), CD31 (-), CD34 (-), SMA (+); Special dyeing results: Elastic fiber (-), iron staining (+), Masson (+), antacid (-). The size of a percutaneous lung biopsy specimen of 1 case was 0.05–0.1 cm. Microscopic findings showed pigmented histopytes, a small amount of cellulose and neutrophils in the alveolar cavity, interscattered with lymphocyte infiltration. Special staining results were as follows: GMS (-), antacid (-), iron staining (+), elastic fiber staining (blood vessels +) (Fig. 3).

## Discussion

In this study, 9 patients with vEDS whose main manifestation was hemoptysis were analyzed. All patients presented with repeated hemoptysis, some with chest pain and liquid pneumothorax and other respiratory symptoms. CT showed that both the two lungs were scattered in the lesion, mainly in the lower lungs, which showed ground glass shadow, fiber cable shadow, nodule and cavity, etc. The ground-glass shadow and nodule could change dynamically, while the other lesions were relatively stable. Pathological examination of 5 patients showed diffuse erythrocyte exudation and hemosiderosis. 8 patients were considered for infectious disease and treated for antibiotic treatment at first diagnosis, and 4 patients even received diagnostic anti-tuberculosis therapy, reflecting the difficulty of hemoptysis vEDS. While pathological findings can provide supportive evidence, genetic testing remains the gold standard for definitive diagnosis and enables family screening.

Although pneumothorax and hemothorax are the more common manifestations of vEDS, hemoptysis is relatively rare. The study by Samia Boussouar showed that 12.5% of patients with pulmonary involvement in vEDS had hemoptysis [10]. Previous literature mainly focused on case reports [11–19], whose CT and pathological features were similar to those in this study, including multiple nodules, cavities and ground-glass shadows, pulmonary vascular destruction, diffuse hemorrhage and increased hemosiderin, and some of them were accompanied by emphysema.

vEDS is a rare autosomal dominant genetic disorder in which a mutation in the COL3A1 gene results in abnormal collagen type III synthesis [23]. Type III collagen is essential for hollow organs, and abnormalities in it can reduce tissue resistance to mechanical stress and cause spontaneous bleeding [8]. In vEDS, decreased respiratory endurance is primarily characterized by dyspnea and limited exercise tolerance, resulting from recurrent spontaneous pneumothorax and hemoptysis. After pulmonary hemorrhage to form a hematoma, through absorption or discharge can form fiber cord or cavity lesions, part of the hematoma calcification to form calcification focus and consolidation appears when infection occurs [10]. The pathological process corresponded to the imaging findings.

Clinically, vEDS is mainly diagnosed by aneurysm, arterial dissection or rupture, intestinal rupture, uterine rupture during pregnancy, family history and genetic testing [9]. Due to the lack of specificity of early symptoms and imaging findings, hemoptysis vEDS is easily misdiagnosed as an infectious disease. Although some patients may improve in the short term after anti-infective therapy, they still experience recurrent hemoptysis, chest pain and CT shows long-term, extensive lesions. Once this occurs, a biopsy of the lung tissue or genetic testing (such as whole exome sequencing or Sanger sequencing test) should be considered to determine whether there is a mutation in the COL3A1 gene, thereby confirming the diagnosis of vEDS. Genetic testing is recommended for a definitive diagnosis. Future multicenter genetic studies involving diverse populations are needed to characterize potential differences in COL3A1 mutation patterns and phenotypic expression between different ethnic groups, which could inform prognosis and management strategies.

Spontaneous arterial dissection, aneurysm, and/or rupture are the most common complications of vEDS [9]. Once these complications occur, vEDS poses a severe threat to the patient’s life. Early diagnosis and appropriate management of vEDS can improve the patient’s prognosis and reduce the occurrence of acute vascular events. Patients should avoid excessive exercise and maintain regular low-intensity exercise to maintain physical and mental health [24]. Studies have shown that long-term administration of angiotensin II receptor blockers and/or beta blockers can reduce the occurrence of vascular events, but there is a lack of randomized controlled trial support [9]. Another study showed that vEDS patients using celiprolol had higher survival rates and lower rates of arterial complications than other treatment groups, but this trial did not include a placebo control group, making it difficult to evaluate the absolute efficacy of celiprolol [5]. Confirmed patients should be checked regularly to monitor the development of the disease. In addition, family members of confirmed patients should consider improving relevant tests.

In conclusion, vEDS should be considered for patients with repeated clinical hemoptysis accompanied by persistent chest CT lesions, and genetic testing should be timely performed to improve patient survival. vEDS should be considered in young men with repeated hemoptysis, multiple, dynamic changes in ground-glass density, fibrous nodules, and cavernous nodules on CT, and no clear evidence of infection or poor efficacy of anti-infective therapy. Genetic testing should be performed as early as possible. However, tissue biopsy, no matter surgical or interventional, should be avoided as far as possible, because of the high possibility in causing severe complications and non-diagnostic pathological findings in patients with vEDS. Timely diagnosis and standardized management are of great significance to improve prognosis.

## Conclusion

This study reports 9 cases of vascular Ehlers-Danlos syndrome (VEDS) with hemoptysis as the main manifestation. CT showed patchy ground glass shadows and cavities in both lungs, and pathology showed diffuse exudation of red blood cells. Due to the lack of specificity, it is often misdiagnosed as an infectious disease. In patients with repeated hemoptysis and persistent CT abnormalities, especially young men, vEDS should be considered and timely genetic testing should be performed. Early diagnosis and standardized management are of great significance for the prevention of serious complications, and it is recommended that relatives of patients should be examined.

## Supplementary Information

Below is the link to the electronic supplementary material.

Supplementary Material 1

Supplementary Material 2

## Data Availability

To safeguard the privacy of study participants, we cannot openly share the data. However, the datasets utilized or analyzed in this study can be obtained from the corresponding author upon reasonable request. Data are stored in controlled access data storage at the First Hospital of Guangzhou Medical University. All extracted data are available upon appropriate requests by emailing to co-corresponding authors.
