# Supplementary material for: Pulmonary vascular Ehlers-Danlos syndrome with hemoptysis as the main manifestation: CT and histologic findings of lung parenchymal damage
Source: Orphanet J Rare Dis. 2025 Nov 21;20:600. doi: 10.1186/s13023-025-04113-4 (PMC12639752; doi:10.1186/s13023-025-04113-4)
Supplement: Supplementary file 1 — Supplementary Material 1 [file 13023_2025_4113_MOESM1_ESM.docx]

STROBE Statement—checklist of items that should be included in reports of observational studies

|  | Item No | Recommendation | Page  No |
| --- | --- | --- | --- |
| **Title and abstract** | 1 | (*a*) Indicate the study’s design with a commonly used term in the title or the abstract | 1 (Title includes "CT and histologic findings") |
|  |  | (*b*) Provide in the abstract an informative and balanced summary of what was done and what was found | 3 (Abstract provides balanced summary of methods and findings) |
| Introduction | | | |
| Background/rationale | 2 | Explain the scientific background and rationale for the investigation being reported | 5-6 (Introduction explains EDS, vEDS, and pulmonary manifestations) |
| Objectives | 3 | State specific objectives, including any prespecified hypotheses | 6 (Lines 109-113 state study aims) |
| Methods | | | |
| Study design | 4 | Present key elements of study design early in the paper | 6-7 (Methods section begins with study design) |
| Setting | 5 | Describe the setting, locations, and relevant dates, including periods of recruitment, exposure, follow-up, and data collection | 7 (Lines 117-126 describe setting and timeframe) |
| Participants | 6 | (*a*) *Cohort study*—Give the eligibility criteria, and the sources and methods of selection of participants. Describe methods of follow-up  *Case-control study*—Give the eligibility criteria, and the sources and methods of case ascertainment and control selection. Give the rationale for the choice of cases and controls  *Cross-sectional study*—Give the eligibility criteria, and the sources and methods of selection of participants | 7 (Lines 117-126 describe eligibility criteria and participant selection) |
|  |  | (*b*) *Cohort study*—For matched studies, give matching criteria and number of exposed and unexposed  *Case-control study*—For matched studies, give matching criteria and the number of controls per case | Not a matched study |
| Variables | 7 | Clearly define all outcomes, exposures, predictors, potential confounders, and effect modifiers. Give diagnostic criteria, if applicable | 7 (Clinical criteria defined in lines 122-126) |
| Data sources/ measurement | 8* | For each variable of interest, give sources of data and details of methods of assessment (measurement). Describe comparability of assessment methods if there is more than one group | *7-8 (Lines 128-138 describe CT protocols, image acquisition, and analysis methods)* |
| Bias | 9 | Describe any efforts to address potential sources of bias | 8 (Clinical data collection method described) |
| Study size | 10 | Explain how the study size was arrived at | 8 (Selection criteria explained in Methods section) |
| Quantitative variables | 11 | Explain how quantitative variables were handled in the analyses. If applicable, describe which groupings were chosen and why | 8 (Lines 140-147 describe CT image analysis) |
| Statistical methods | 12 | (*a*) Describe all statistical methods, including those used to control for confounding | 8-9 (Descriptive statistical methods mentioned) |
|  |  | (*b*) Describe any methods used to examine subgroups and interactions | No subgroup analyses |
|  |  | (*c*) Explain how missing data were addressed | No missing data reported |
|  |  | (*d*) *Cohort study*—If applicable, explain how loss to follow-up was addressed  *Case-control study*—If applicable, explain how matching of cases and controls was addressed  *Cross-sectional study*—If applicable, describe analytical methods taking account of sampling strategy | No loss to follow-up reported |
|  |  | (*e*) Describe any sensitivity analyses | No sensitivity analyses performed |

Continued on next page

| Results | | | |
| --- | --- | --- | --- |
| Participants | 13* | (a) Report numbers of individuals at each stage of study—eg numbers potentially eligible, examined for eligibility, confirmed eligible, included in the study, completing follow-up, and analysed | 8 (Lines 158-162 describe selection of included patients) |
|  |  | (b) Give reasons for non-participation at each stage | 8 (Exclusion criteria described in lines 160-162) |
|  |  | (c) Consider use of a flow diagram | No flow diagram included |
| Descriptive data | 14* | (a) Give characteristics of study participants (eg demographic, clinical, social) and information on exposures and potential confounders | 8-9 (Table 1 and lines 162-164 describe participant characteristics) |
|  |  | (b) Indicate number of participants with missing data for each variable of interest | All data appears complete |
|  |  | (c) *Cohort study*—Summarise follow-up time (eg, average and total amount) | 9 (Line 164 reports follow-up time) |
| Outcome data | 15* | *Cohort study*—Report numbers of outcome events or summary measures over time | *9-10 (Results of clinical manifestations and CT features reported)* |
|  |  | *Case-control study—*Report numbers in each exposure category, or summary measures of exposure | All data appears complete |
|  |  | *Cross-sectional study—*Report numbers of outcome events or summary measures | All data appears complete |
| Main results | 16 | (*a*) Give unadjusted estimates and, if applicable, confounder-adjusted estimates and their precision (eg, 95% confidence interval). Make clear which confounders were adjusted for and why they were included | 9-11 (Results presented as descriptive findings without adjustment) |
|  |  | (*b*) Report category boundaries when continuous variables were categorized | No categorization of continuous variables |
|  |  | (*c*) If relevant, consider translating estimates of relative risk into absolute risk for a meaningful time period | Not a risk estimation study |
| Other analyses | 17 | Report other analyses done—eg analyses of subgroups and interactions, and sensitivity analyses | No additional analyses performed |
| Discussion | | | |
| Key results | 18 | Summarise key results with reference to study objectives | 12 (Lines 233-242 summarize key findings) |
| Limitations | 19 | Discuss limitations of the study, taking into account sources of potential bias or imprecision. Discuss both direction and magnitude of any potential bias | 12-13 (Discussion acknowledges rarity of presentation) |
| Interpretation | 20 | Give a cautious overall interpretation of results considering objectives, limitations, multiplicity of analyses, results from similar studies, and other relevant evidence | 12-14 (Discussion compares findings to previous studies and literature) |
| Generalisability | 21 | Discuss the generalisability (external validity) of the study results | 14 (Limitations recognized as a rare presentation) |
| Other information | | | |
| Funding | 22 | Give the source of funding and the role of the funders for the present study and, if applicable, for the original study on which the present article is based | 2 (Funding information provided) |

*Give information separately for cases and controls in case-control studies and, if applicable, for exposed and unexposed groups in cohort and cross-sectional studies.

**Note:** An Explanation and Elaboration article discusses each checklist item and gives methodological background and published examples of transparent reporting. The STROBE checklist is best used in conjunction with this article (freely available on the Web sites of PLoS Medicine at http://www.plosmedicine.org/, Annals of Internal Medicine at http://www.annals.org/, and Epidemiology at http://www.epidem.com/). Information on the STROBE Initiative is available at www.strobe-statement.org.
