# Supplementary material for: Pulmonary vascular Ehlers-Danlos syndrome with hemoptysis as the main manifestation: CT and histologic findings of lung parenchymal damage
Source: Orphanet J Rare Dis. 2025 Nov 21;20:600. doi: 10.1186/s13023-025-04113-4 (PMC12639752; doi:10.1186/s13023-025-04113-4)
Supplement: Supplementary file 2 — Supplementary Material 2 [file 13023_2025_4113_MOESM2_ESM.docx]

**Supplemental Table. Chest CT Features of Pulmonary Hemoptysis-Type vEDS Patients**

| Case | Location | GGO | Con | LO | CN | NCN | CalN | E/B | P/H |
| --- | --- | --- | --- | --- | --- | --- | --- | --- | --- |
| 1 | Bil | + | + | + | + | + | - | - | - |
| 2 | Bil | + | - | + | + | + | - | - | - |
| 3 | Bil | + | - | + | + | + | - | - | - |
| 4 | Bil | + | + | + | + | + | + | + | + |
| 5 | Bil | + | - | + | - | + | + | + | + |
| 6 | Bil | + | + | - | + | + | - | + | - |
| 7 | RUL & BLL | + | - | + | - | + | - | - | - |
| 8 | BLL | + | - | + | + | + | - | - | - |
| 9 | Bil | + | - | + | + | + | - | - | - |

Abbreviations:

Bil = Bilateral; BLL = Bilateral Lower Lobes; RUL = Right Upper Lobe; GGO = Ground Glass Opacity; Con = Consolidation; LO = Linear Opacity; CN = Cavitary Nodule; NCN = Non-Cavitary Nodule; CalN = Calcified Nodule; E/B = Emphysema/Bullae; P/H = Pneumo/Hydropneumothorax
